# Peptide-based coatings for flexible implantable neural interfaces

**DOI:** 10.1038/s41598-017-17877-y

**Published:** 2018-01-11

**Authors:** Martina Righi, Gian Luigi Puleo, Ilaria Tonazzini, Guido Giudetti, Marco Cecchini, Silvestro Micera

**Affiliations:** 1 The BioRobotics Institute, Scuola Superiore Sant’Anna, Viale Rinaldo Piaggio 34, 56025 Pontedera (PI), Italy; 2 Istituto Italiano di Tecnologia, Center of Micro-BioRobotics@SSSA, Viale Rinaldo Piaggio 34, 56025 Pontedera (PI), Italy; 3 NEST (National Enterprise for nanoScience and nanoTechnology), Istituto Nanoscienze-CNR & Scuola Normale Superiore, Piazza San Silvestro 12, 56127 Pisa, Italy; 4 Bertarelli Foundation Chair in NeuroEngineering, Center for Neuroprosthetics and Institute of Bioengineering (IBI)-School of Engineering, École Polytechnique Fédérale de Lausanne (EPFL), Lausanne, Switzerland

## Abstract

In the last decade, the use of flexible biosensors for neuroprosthetic and translational applications has widely increased. Among them, the polyimide (PI)-based thin-film electrodes got a large popularity. However, the usability of these devices is still hampered by a non-optimal tissue-device interface that usually compromises the long-term quality of neural signals. Advanced strategies able to improve the surface properties of these devices have been developed in the recent past. Unfortunately, most of them are not easy to be developed and combined with micro-fabrication processes, and require long-term efforts to be testable with human subjects. Here we show the results of the design and *in vitro* testing of an easy-to-implement and potentially interesting coating approach for thin-film electrodes. In particular, two biocompatible coatings were obtained via covalent conjugation of a laminin-derived peptide, CAS-IKVAV-S (IKV), with polyimide sheets that we previously functionalized with vinyl- and amino- groups (PI_v and PI_a respectively). Both the engineered coatings (PI_v+IKV and PI_a+IKV) showed morphological and chemical properties able to support neuronal adhesion, neurite sprouting, and peripheral glial cell viability while reducing the fibroblasts contamination of the substrate. In particular, PI_v+IKV showed promising results that encourage further *in vivo* investigation and pave the way for a new generation of peptide-coated thin-film electrodes.

## Introduction

Neurological disorders, nervous system injuries, and limb amputations generally cause impairments, which result in a highly disabling impact and markedly affect the quality of life of individuals^1^.

For these reasons, in the recent past, the challenge of restoring sensory-motor functions has received increasing attention from engineers, neurophysiologists, and clinicians. Neural interfaces^2^, able to record high quality signals and to properly stimulate the nervous system, for the delivery of sensory feedback, have been recognized as the key component to re-establish an “intimate”, functional and bidirectional electrical connection with the nervous system of an impaired patient^3,4^. The development of personalized neuroprostheses^5^ for specific tasks and applications encouraged the manufacturing of new and effective neural interfaces and the investigation of different materials and fabrication techniques^6–11^.

In particular, polyimide has recently gained wide popularity as high-performance material for neural implants and applications. Thanks to some excellent characteristics (such as biocompatibility, low water uptake, high mechanical strength, and dielectric properties^12^), it has been used as coating for several invasive neural electrodes^6–9,13,14^.

Unfortunately, notwithstanding important efforts carried out in the past, current neural interfaces in general and polyimide-based electrodes in particular are still not able to achieve stable and optimal long-term performance after the implantation^13^. The invasiveness of these devices, despite fundamental to increase their level of selectivity, induces neural tissue damages and axonal injuries, with consequent local inflammation and immune system reactions that frequently result in the rejection or insulation of the device from the neural tissue. The foreign-body response is mainly characterized by a large astrocytes activation in the central nervous system (CNS), while in the peripheral one (PNS) it involves macrophages recruitment and a consequent formation of a dense and compact layer of fibroblasts and collagen^14^ which limits the tissue repair response of Schwann cells following a peripheral nervous system injury^15–17^. This reaction causes the encapsulation of electrodes and hampers axonal re-growth, culminating in variability of stimulation efficiency and lack of recording reproducibility. Therefore, one of the biggest challenges is to reduce the mismatch between the device and the tissue of implantation using proper coatings. In particular, besides the biocompatibility of the employed materials, the structural and surface characteristics of the selected coatings are essential requirements to support axonal viability and to limit the fibroblast contamination of an invasive neural interface^18–20^. Furthermore, the suitability of different approaches and the use of specific techniques strongly influence the applicability of the device to specific targets. In particular, a high complexity of the coating structure and/or the use of novel and purposely-designed materials^21^, despite being interesting from a scientific viewpoint, usually hamper swift usability of such a device for translational applications. Previous works demonstrated how surface topography, rigidity, wettability and the presence of bioactive molecules or proteins influence the adhesion, proliferation and differentiation of cells^22–25^. In particular, laminin (a multidomained glycoprotein of the extracellular matrix) is able to support neuron development, migration and neurite outgrowth during embryonic development and tissue regeneration^26,27^ and has a crucial role in PC12 cells adhesion^28^, neural differentiation and axonal growth, being involved in the interaction with receptors at cell surface (such as integrins of the β family, non-integrin binding proteins and carbohydrate-binding moieties such as lectins or galactosyltransferase). Additional studies also demonstrated that laminin can enhance the chronic recording stability of silicon microelectrode arrays in the CNS^29,30^, encouraging the use of this protein in invasive devices for neural applications.

The main aim of this work was to use a laminin-derived peptide to engineer a simple, biomolecule-based coating for polyimide thin-film electrodes for peripheral nervous system applications. This approach, suitable and easily implementable into the fabrication process of these devices, was conceived to improve polyimide surface properties, and especially addressed to increase neural support and Schwann cells response, while limiting fibroblast adhesion.

In particular, via the conjugation of the laminin-derived peptide CAS-IKVAV-S (IKV) to the vinyl- and amino- groups of functionalized polyimide sheets (PI_v and PI_a), we developed and tested two different modification strategies (PI_v+IKV and PI_a+IKV), suitable and adaptable for thin-film polyimide electrodes production^6–9^, and easily implementable in their microfabrication process. The use of a peptide can reduce the issues related to the steric accessibility^31^ of a surface-bound whole protein: the IKVAV sequence was selected among others (i.e. the RGD sequence) because it is known to support neuron adhesion and growth onto a substrate, while limiting the fibroblasts ones^32^.

The measured morphological (roughness and wettability) and chemical properties of both PI_v+IKV and PI_a+IKV, and *in vitro* tests, confirmed an increased biocompatibility of the material and a reduction in the fibroblast contamination of the substrates, compared to neural-like cell lines. In particular, PI_v+IKV demonstrated to strongly support the adhesion, differentiation and sprouting of neuronal and peripheral glial cells (PC12 and primary Schwann cells, respectively) while not significantly contributing in terms of fibroblast response to the surface. These results lead to consider PI_v+IKV as a promising technical solution for chronic peripheral implants, and open up the way for further *in vivo* testing.

## Methods

### Development of polyimide films

Polyimide (PI) films were produced using thin-film technology. Shortly, polyimide resin PI 2610 (Hitachi Chemical DuPont MicroSystems GmbH) was spin-coated at 10000 rpm for 30 sec (thickness = 700 nm) onto a 6 mm diameter polished glass disc previously cleaned with acetone (GLC 99,8%) and isopropanol (GLC 99,9%) in order to remove organic contaminants or polluting substances. After the spinning, the samples were soft-baked at 130 °C on a hot plate and subsequently cured at 350 °C in nitrogen atmosphere for 60 min (hard-bake)^33^.

### Surface covalent modification of polyimide films

#### Methacrylamide-functionalized samples (PI_v)

Polyimide films functionalization was obtained as reported^34^. Shortly, the samples (d = 6 mm each) were treated overnight at room temperature in 2 mL of methanol in the presence of 20 mg (0.11 mmol) N-(3-Aminopropyl)methacrylamide hydrochloride (Polysciences) and 0.5 mL (2 mmol) tributylamine (Sigma Aldrich) in the dark, in order to obtain methacrylamide-modified polyimide samples (PI_v). The samples were then washed twice with methanol and distilled water and let dry at room temperature overnight.

#### Amino-functionalized samples (PI_a)

In order to introduce amino functional groups upon polyimide films (PI_a), PI samples were incubated overnight at room temperature in 1.5 mL of methanol in presence of 1.5 mL of ethylendiamine (Sigma Aldrich)^35^. Samples were finally washed twice with methanol and distilled water and let dry at room temperature overnight.

### Preparation of peptide-based PI_v+IKV

**Figure 1:**
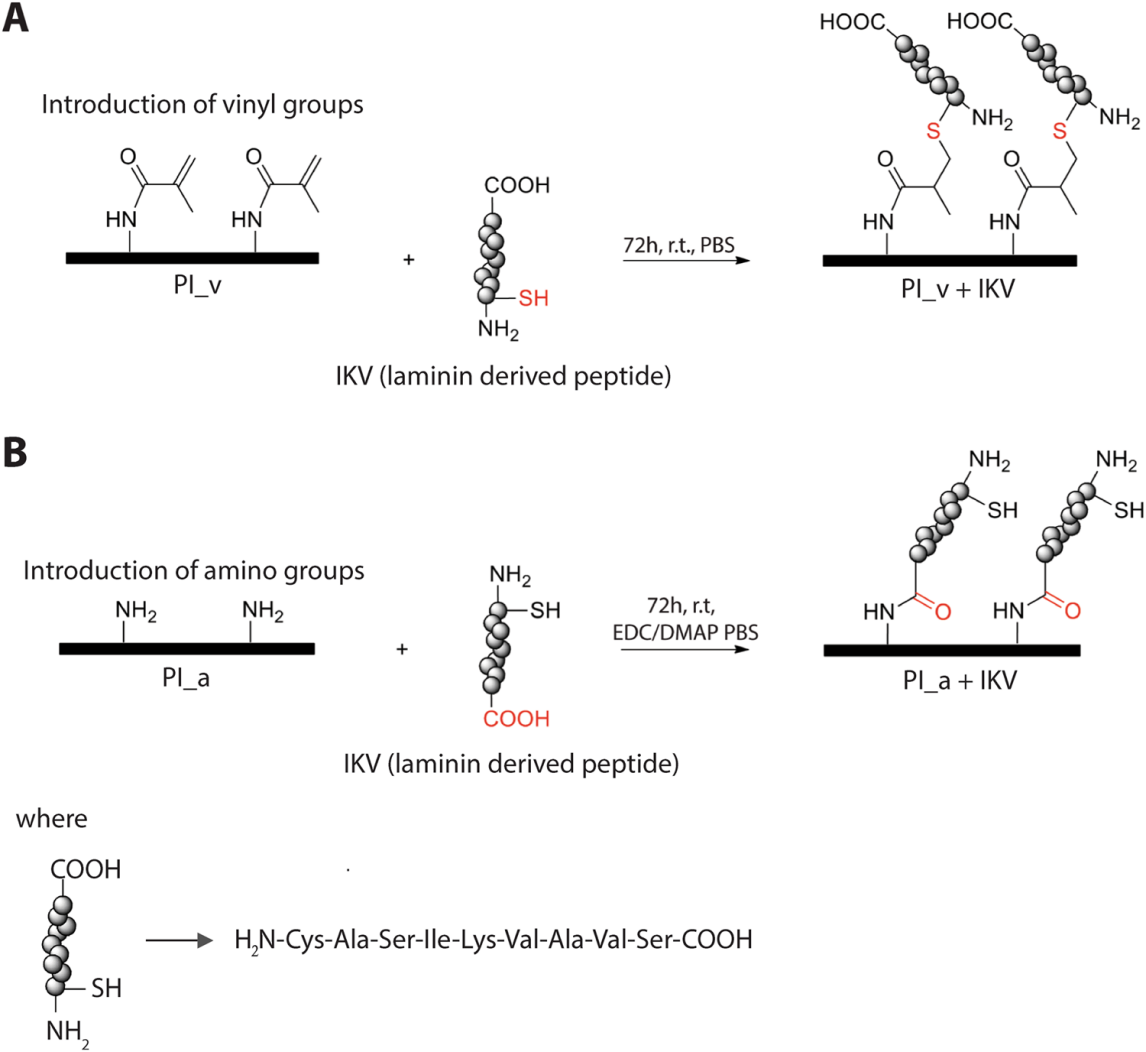
Schematization of two different strategies for the development of a peptide-coated polyimide film (PI). (**A**) Conjugation of the IKV peptide to a methacrylamide-functionalized PI sample via Michael-type addition. (**B**) Conjugation of the IKV peptide to an amino-functionalized sample via standard peptide bonding reaction. In this case the covalent conjugation with the amino groups upon PI_a was obtained after the activation of the peptide with the EDC/DMAP reactive solution.

Two suspensions, respectively 1.2 mM of a custom synthetic peptide CAS-IKVAV-S (IKV) (ThermoFisher Scientific) and 1.2 mM of tris(2-carboxyethyl)phosphine TCEP (Sigma Aldrich), were prepared in a phosphate buffer solution (PBS, pH 7.4), mixed in equal amount (1:1) and heated at 40 °C for 45 min to reduce the peptides disulphide bonds (S-S). PI_v samples were then treated with this solution for 72 h at room temperature, and kept in gentle shaking in the absence of light. After that, the solution was removed and samples were washed first with PBS in order to eliminate unreacted peptides and subsequently with deionized water (DI water) to remove excess PBS salts. To avoid bio-contamination, all the samples were stored overnight at 4 °C in a solution of PBS (pH 7.4) and antibiotics (100 IU/mL penicillin, 100 µL/mL streptomycin) before cells seeding (Fig. 1A).

### Preparation of peptide-based PI_a+IKV

A 1.2 mM solution of synthetic IKV peptide was prepared and mixed with a 1.2 mM TCEP solution as previously described. In parallel, a 1.2 mM reactive solution was prepared dissolving 1-Ethyl-3-(3-dimethylaminopropyl)carbodiimide (EDC) and 4-Dimethylaminopyridine (DMAP) (Sigma Aldrich)^36^ in PBS (pH 7.4). A 1:10000 diluted solution was then mixed in equal amount (1:1) with the peptide/TCEP one and stored for 1 h at room temperature (Fig. 1B). A large excess of activated IKV was used to cap all the amino-groups exposed on the PI_a surface and thus obtain an effective peptide conjugation.

Also in this case, PI_a samples were treated with this solution for 72 h at room temperature and kept in gentle shaking in the absence of light. After that, the solution was removed and samples were washed and stored as previously described before cells seeding.

### Surface characterization of modified polyimide films

#### Atomic Force Microscopy (AFM) measurements

Atomic force microscopy measures were performed with an Innova SPM Scanning Probe Microscope (Bruker Corporation, Germany) equipped with SPMLab Vr. 5.01 software, in tapping mode in air with a scan range of 5 µm and a scan rate of 0.5 Hz. Images were analysed with the Gwyddion software (Czech Metrology Institute, general public license)^37^. Changes on surface morphology were quantified by root-mean-square (Rms) roughness values (Rq) defined as in Equation 1:

1 $${{\boldsymbol{R}}}_{{\boldsymbol{q}}}=\sqrt{\frac{{\sum ({Z}_{i}-{Z}_{avg})}^{2}}{{\boldsymbol{N}}}},$$

where Z_avg_ is the average Z height value within a given area, Z_i_ is the current Z value, and N is the number of points within the given area.

The roughness was measured at 5 different and arbitrary areas for each sample to calculate means and standard deviation (SD) and untreated polyimide was used as reference control.

#### Contact Angle measurements

The wettability of the substrates was evaluated using the sessile drop method by means of an Attension Theta optical tensiometer (Biolin Scientific). A droplet of 2 µL of deionized water was deposited upon the sample (d = 6 mm) and the spreading of the droplet was imaged at 10 frames/sec for a total range of 20 sec. The angles were measured at 3 different and arbitrary areas for each sample to calculate means and standard deviation (SD) and the untreated polyimide was used as reference control.

#### Fourier Transform Infrared Spectroscopy (FT-IR) measurements

Infrared spectra of samples were taken in transmittance mode (T%) by means of an IRPrestige-21 IRAfinity-1 FTIR-8400S (Shimadzu, Japan). Measurements were performed with a spectral range of 750–4000 cm^−1^ by accumulation of 200 scans and a resolution of 4 cm^−1^.

The IR spectra of functionalized samples were analyzed using the dedicated software (Omni Spectra Software) and compared with the untreated polyimide peak bands, used as reference control. For each specimen 3 measurements were performed, each one in a different and arbitrary position within the total area.

#### X-ray Photoelectron Spectroscopy (XPS) measurements

The surface chemical composition of untreated and differently functionalized polyimide films was determined with X-Ray Photoelectron Spectroscopy (XPS) measurements using a PHI VersaProbe II scanning XPS microprobe (Physical Instruments AG, Germany). All the measurements were performed using a monochromatic Al Kα X-ray source of 24.8 W power with a beam size of 100 µm. Spectra were acquired with a pass energy of 46.95 eV yielding a full width at half maximum of 0.91 eV for the Ag 3d 5/2 peak. Curve fitting was performed using the PHI Multipak software, the binding energy was calibrated by the C1s peak at 284.84 eV and peak area and peak height sensitivity factors were used for the quantifications of different elements atomic concentration. As for the previous analysis, 3 measurements were performed on each specimen, each one on a different position on the total area.

### *In vitro* test methods

#### PC12 cells culture

Transplantable rat pheochromocytoma derived PC12 cells (ATCC CRL- 1721) were seeded on samples and controls (untreated glass coverslips as negative control and collagenated multiwell plates as positive control) and cultured in Dulbecco’s Modified Eagle medium (DMEM)-high glucose supplemented with 10% fetal bovine serum (FBS), 5% horse serum (HS), 100 IU/mL penicillin, 100 mg/mL streptomycin and 2 mM L-Glutamine. Cells were maintained at 37 °C in a saturated humidity atmosphere and 5% CO_2_ as previously described^38,39^.

Each sample was laid in a single well of a 24 wells plate. Two different experiment conditions were set up in order to evaluate cell adhesion and cell differentiation. For both these conditions, at least 3 replicate experiments were performed, each one consisting of 3 technical replicates for each substrate. For adhesion analysis cells were seeded with a density of 20000 cells/cm^2^ and fixed after 24 h.

For differentiation experiments, cells were seeded at a density of 12500 cells/cm^2^. After 24 h, the standard growth medium was replaced with a differentiating one, containing 1% FBS, 100 IU/mL penicillin, 100 µL/mL of streptomycin, 2 mM L-Glutamine and 60 ng/mL nerve growth factor (NGF-β human, N1418, Sigma). PC12 cells were grown for further 7 days and finally fixed and stained as described in the following.

#### Fibroblasts culture

Human dermal fibroblasts (NHDF) (code: CC 2511, Lonza USA) were seeded on samples and controls (glass as negative and multiwell plate as positive ones) and cultured in DMEM high glucose supplemented with 10% fetal bovine serum (FBS), 100 IU/mL penicillin, 100 mg/mL streptomycin and 2 mM L-Glutamine. Cells were maintained at 37 °C in a saturated humidity atmosphere and 5% CO_2_ as previously described^40^. As for PC12 tests, at least 3 replicate experiments were performed, each one consisting of a triplicate of substrates. Each sample was settled in a single well of a 24 wells plate, cells were seeded at 20000 cells/cm^2^ and fixed after 24 h.

#### Cell Fixation and Nuclei Staining

After 24 h or 7 days of incubation (depending on the test), the medium was removed and samples were washed with PBS 1X. Fixation was performed with 4% paraformaldehyde in PBS for 15 min, then the fixed cells were washed three times with PBS 1X (5 min for each wash) and treated with 0.1% Triton X-100 in PBS for 15 min. After that, fixed cells were washed 2 times with PBS 1X (5 min each wash) and incubated (15 min) at room temperature with 4′,6-diamidino-2-phenylindole (DAPI) 1 µL/mL in PBS to stain nuclei. Finally, cells were washed with PBS 1X (5 min) and observed with an epifluorescence microscope (ECLIPSE Ti-E Inverted epifluorescence microscope, Nikon Instruments). Pictures were taken at 10X magnification using a DAPI filter (440 nm) acquired with a high-resolution device camera and processed by means of the dedicated NisElements software (Nikon).

The Fiji software^41^ (an implementation of ImageJ by the U.S. National Institutes of Health) was used to quantify the cell density of each sample, by counting the number of DAPI-stained nuclei present in each micrograph of 5 different and arbitrary areas per sample. Data were reported as mean and standard error of the mean (SEM).

#### Quantification of neurite outgrowth

After 7 days cells were fixed and stained as previously described. In this case, TRITC conjugated phalloidin 1 µL/mL was added to the DAPI solution to stain actin fibers and nuclei, respectively. Cells were observed with the epifluorescence microscope at 20X magnification and pictures taken using apt filters, specific for DAPI (440 nm) and TRITC (605 nm) respectively.

PC12 neuronal morphology was analysed by two different parameters: the mean neurite length per cell (defined as the sum of all the cell protrusions on each image, mediated on the total number of differentiated cells per image) and the maximum neurite sprouting (the single longest cell protrusion per image, in micrometers) were quantified by means of NeuronJ^42^, an ImageJ plugin, used to trace neurites.

We defined a PC12 as a differentiated cell by morphology, when it showed at least one neurite originating from the cell body: only protrusions longer than 10 µm (about one average cell body diameter) were counted as neurites^43^. Data were reported as mean and SEM, as previously described.

#### Schwann cell culture

Primary SC cultures were established from sciatic nerves of adult Wistar rats^44,45^. SCs were maintained in the presence of glial growth factor (GGF 63 ng/mL; SRP3055, Sigma) and Forskolin (10 µM; F3917, Sigma) in DMEM medium supplemented with 10% fetal bovine serum, 4 mM L-glutamine, and antibiotics, in Poly-D-Lysine coated (100 µg/mL; P0899, Sigma) standard culture plates. Cells were routinely immunodepleted by anti-rat Thy1.1 antibody (1:500, MCA04G; AbD Serotec) to reduce the presence of fibroblasts. SCs (within the 11^th^ passage) were cultured until sub-confluence, and then harvested for cell tests.

#### Schwann cell adhesion and proliferation assays

SCs were seeded on different substrates (placed in 96 well-plates) at a density of 2.3*10^4^ cells/cm^2^, for different times (2–72 h), under standard cell culture conditions.

SCs short-term adhesion (2–6 h) was investigated by bright-field imaging. An inverted microscope Leica DMI 4000 B (Leica Microsystems, Wetzlar, Germany) was used to monitor cells. Pictures were taken in 5 different and arbitrary sites for each sample and the cell quantity was obtained using the ImageJ software (U.S. National Institutes of Health).

SC cell viability (24 h) and proliferation (72 h) were measured by the 2-(2-methoxy-4-nitrophenyl)-3-(4-nitrophenyl)-5-(2,4-disulfophenyl)-2H-tetrazolium-monosodium salt (WST-8) assay, according to instructions (Sigma, #96992). After 24 and 72 h from seeding, SCs were incubated in a 10% WST-8 solution (in medium) in a CO_2_ incubator for 3 h. Afterwards, the supernatant was carefully aspirated, transferred to a new plate, and the absorbance of each well was observed by a plate reader at a wavelength of 450 nm. The absorbance of formazan produced is directly proportional to the number of living cells. As a reference, the density of SCs on PI substrate at 24 h was 81 ± 9% in respect to a standard plastic well plate and 125 ± 10% in respect to a glass coverslip.

As for the other cell types, data were reported as mean and standard error of the mean (SEM); at least 3 replicate experiments were performed for each condition.

#### Immunostaining, confocal imaging and cell morphological analysis

SCs were grown for 3–4 days on different substrates, then processed for immunostaining as previously reported^45^. Briefly SCs were fixed in 4% paraformaldehyde and stained with anti-S100 primary antibody (Sigma; 1:200, rabbit) and phalloidin-AlexaFluor647 (Invitrogen; 1:40) in GDB buffer (0.2% BSA, 0.8 M NaCl, 0.5% Triton X-100, 30 mM phosphate buffer, pH 7.4) overnight at 4 °C. Samples were then washed and incubated with the AlexaFluor488-conjugated secondary antibody (Invitrogen; 1:150, anti-rabbit) in GDB for 2 h at room temperature. After washing, samples were mounted using Fluoroshield mounting medium with DAPI to stain nuclei (Sigma).

Confocal images were acquired using a laser scanning confocal microscope TCS SP2 (Leica Microsystems, Germany) with a 40× oil objective, by using UV (405 nm) and argon (488 nm) lasers. Each reported confocal image was obtained from a *z*-series (stack-depth was around 6 µm; steps = 1 µm). The resulting *z*-stack was processed by the ImageJ software into a single image using the *z-project* and *Max intensity* options^45^. The confocal images of S100-staining (specific SC marker) were used to evaluate cellular morphology by ImageJ. Cell contours were drawn by the *Free-hand selection* tool and processed by the *Measurement* tool (selecting the options *Area* and *Feret’s diameter*). The parameters measured in this analysis were: SC *area* (μm^2^) and the SC *elongation ratio* (the ratio between the longest length of the cell along the *major axis* (μm) and the width of the cell perpendicular to the major axis)^46^.

SC sprouting was also quantified. The protrusions of each cell were semi-automatically segmented (from the point of origin on the cell body to their tip) on the confocal Z-stack images using NeuronJ. A file containing the tracks was exported and loaded into Matlab (MathWorks) to calculate the protrusion length (the distance of the traced path, in μm)^47^. The number of protrusions/cell was also quantified; segments with length < 10 µm were excluded from this analysis.

### Statistical Analysis

Data were statistically analyzed by using the commercial software GraphPad Prism (San Diego, CA, USA). The mean values obtained in each repeated experiment were assumed to be normally distributed around the true mean. One-Way ANOVA (Dunnett’s multiple comparison test) analysis was used, unless otherwise stated, to compare substrates to the PI substrate; alternatively the Student *t*-test (two-tailed, unpaired) was performed to directly compare different substrates. Statistical significance refers to results where *P* < 0.05 was obtained.

## Results

The functionalization of a polyimide film surface (concept described in Fig. 1) allowed us to design and successfully manufacture two advanced peptide-based biomaterials for invasive thin-film electrodes (PI_v+IKV and PI_a+IKV). The bioactive molecule (IKV) was composed by the laminin-derived IKVAV sequence (specifically selected to improve the material properties at the interface with neurons, Schwann cells and fibroblasts^32^) endowed with a final cysteine (C) able to interact with the reactive groups exposed on the functionalized PI. The peptide immobilization was obtained by two different strategies that allowed the stable covalent bonding between the peptide and the polymer.

For the PI_v+IKV coating (Fig. 1A), the cysteine moiety at the end of the IKV chain was involved in a Michael-type addition^48^ between the thiol-groups of this aminoacid and the vinyl-groups exposed on the PI_v surface, ensuring a stable covalent bond. A previous report by our group^49^ already demonstrated how to successfully establish a covalent conjugation between a thiol-containing amino acid (L-Cysteine functionalized with Rhodamine B) and a PI_v film thanks to the 1,4-addition of the nucleophilic sulphur of the cysteine and the electrophilic double bond of the unsaturated amide.

Differently, to obtain the PI_a+IKV coating, our strategy (Fig. 1B) was based on the covalent conjugation between IKV and PI_a by a standard peptide bond (-CONH-) cross-coupling. In particular, the IKV carboxylic acid terminus was activated by an appropriate cross-coupling reagent, the EDC/DMAP solution, to promote the reaction with the amino groups exposed on the PI_a surface^36^.

For both procedures, the covalent conjugation steps of IKV were preceded by the reduction of peptide disulfides to thiol groups by using TCEP, a kinetically stable reagent in aqueous solutions, selective towards disulfide links and unreactive to the other functional groups^50^.

Surface analysis, performed to investigate morphology, wettability and chemical composition of samples, was combined with *in vitro* testing to assess the biomaterials interaction with different types of cells (PC12, primary Schwann cells, fibroblasts). PI_v+IKV and PI_a+IKV were first compared with untreated PI (used as negative control) and subsequently with PI_v and PI_a.

### Surface characterization of peptide-based polyimide sheets

To assess whether and how treatments and functionalizations affected the morphology and chemical composition of the polymer, different surface analyses - atomic force microscopy (AFM), static contact angle measurement (SCA), Fourier transform infrared spectroscopy (FT-IR) and X-ray photoelectron spectroscopy (XPS) - were performed. Each sample was scanned in multiple and arbitrary spots of the total area and the mean values of the outcomes were used for statistics. Moreover, results for PI and PI_v related to previous works^34,49^ were used as reference.

**Figure 2:**
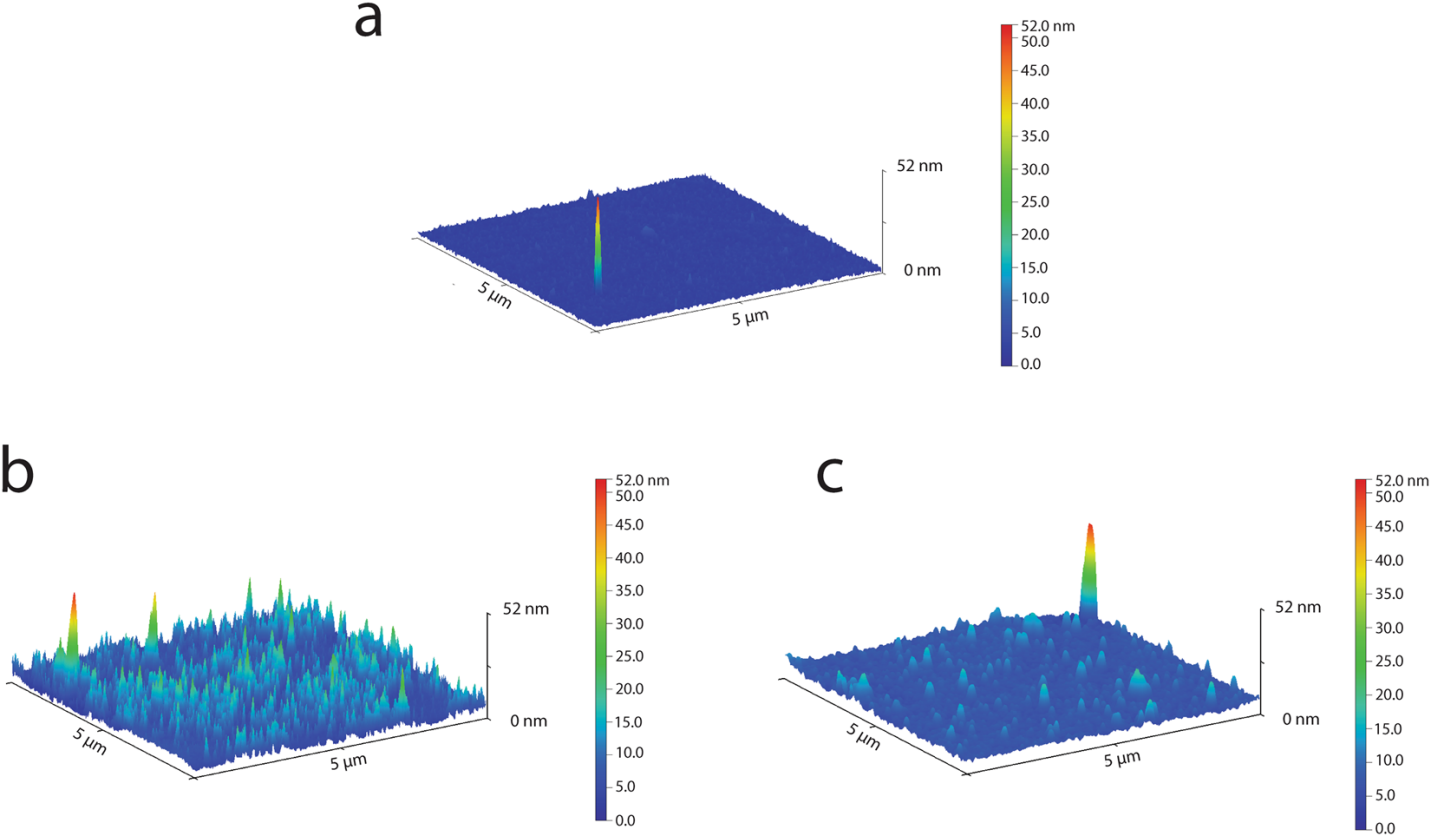
AFM 3D topography of samples surfaces. Scans with an area of 5 × 5 µm were performed in tapping mode for each sample with a scan rate of 0.5 Hz: untreated PI (**a**) was used as control to evaluate PI_v+IKV (**b**), and PI_a+IKV (**c**). Rms was calculated to quantitatively estimate the entity of surface modification introduced with chemical functionalizations and IKV peptide conjugation.

Unmodified PI showed a smooth surface, with no features or irregular areas, with a root-mean-square roughness value (R_ms_) of ~0.57 nm and a contact angle of ~63°. Conversely, a large number of nanoscale depressions (Fig. 2) characterized both PI_v and PI_a, and markedly influenced R_ms_ and wettability outcomes. Table 1 shows, by contact angle measurements, how the introduction of vinyl- groups caused a hydrophobic effect, while the amino- one contributed to enhance the wettability of the material.

**Table 1:** Surface roughness (Rms) and static contact angle (SCA) measurements for PI and modified PI samples. Results are reported as mean and SD.

	PI	PI_v	PI_a	PI_v+IKV	PI_a+IKV
AFM [Rms; nm]	0,57 ± 0,03	2,39 ± 0,79	3,93 ± 0,34	3,15 ± 0,94	4 ± 0,80
SCA [°]	65 ± 8,17	70 ± 5,17	61 ± 4,65	59 ± 11,66	58 ± 4,67

Finally, the IKV conjugation showed to just barely contribute in terms of roughness to both approaches, but it was responsible for a relevant increase of PI_v+IKV wettability (Table 1).

In order to confirm the correlation between these morphological changes and the presence of functional groups and/or biomolecules on the polyimide surface, FT-IR analysis and XPS were performed. The resonant frequencies associated to the specific vibration of molecular moieties contained in surface modifiers allowed to characterize their presence on polyimide surface.

The results, obtained from the comparison of PI_v and PI_a with the untreated polyimide, revealed the presence of a peak in correspondence of 3069 cm^−1^ and 3071 cm^−1^ respectively, and a consequent modification of the entire spectrum (Supplementary Figure S1). In particular, these outcomes evidenced the presence of the surface modifiers and can be attributed to the antisymmetric stretching of CH_2_(CH = CH_2_) in vinyl- groups and to the stretching of N-H in amino-groups. This data, obtained from the analysis of 3 replicates for each sample type and the measurements of 3 arbitrary spots/sample, confirmed the repeatability of our results. Nevertheless, no evidence of additional changes was observed after the introduction of the laminin-derived peptide (PI_v+IKV and PI_a+IKV). One of the possible explanations is the overlapping of its molecular vibration patterns with the ones of PI_v or PI_a.

**Figure 3:**
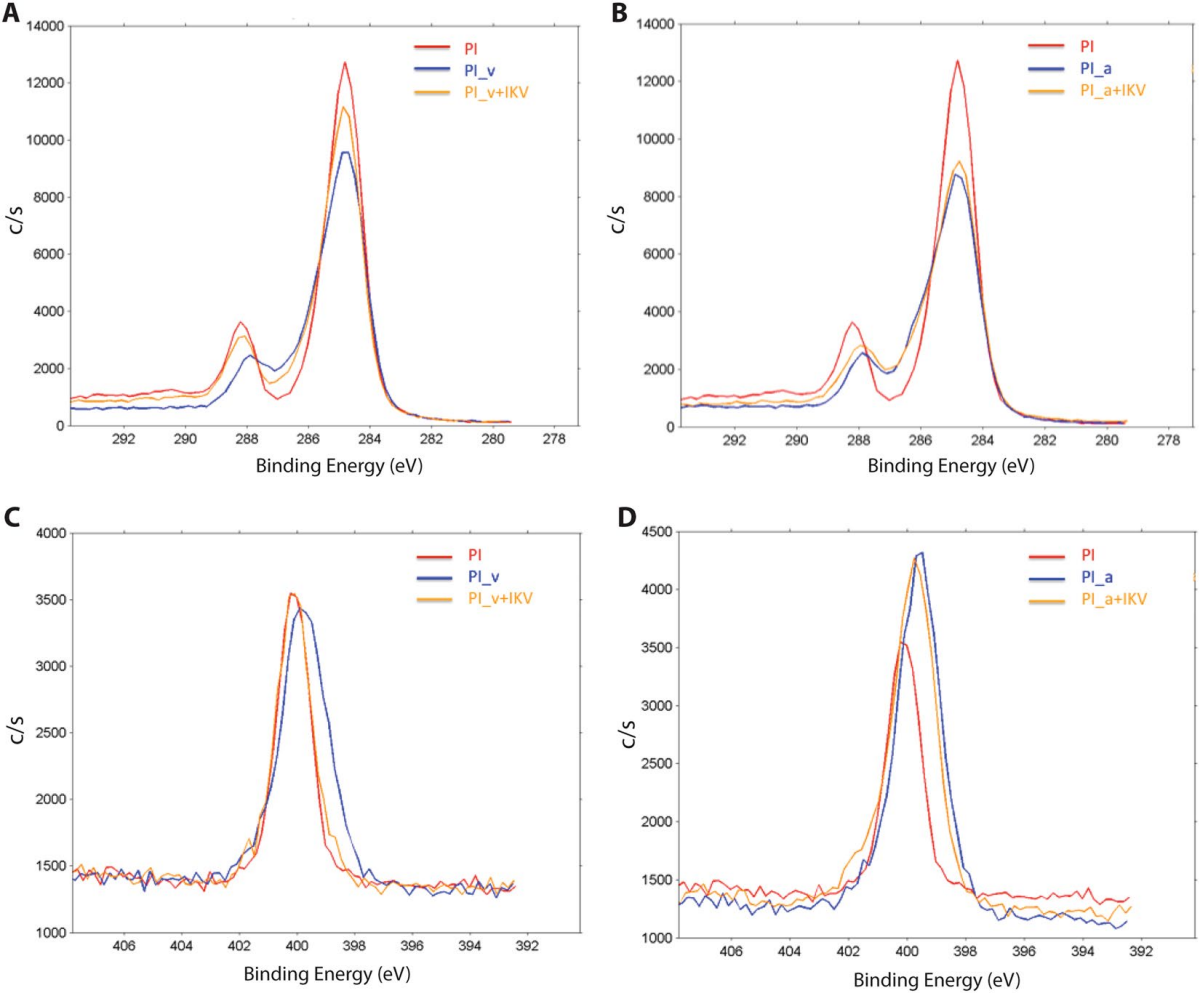
XPS analysis on samples surfaces with Carbon (**A**,**B**) and Nitrogen (**C**,**D**) peaks and curves. As in the other tests, untreated PI was used as negative control and its surface composition compared with those of PI_v and PI_v+IKV (**A**,**B**) and with PI_a and PI_a+IKV (**C**,**D**).

The quantitative evaluation of the surface chemical composition was performed for each sample with XPS measurements. The atomic concentrations of carbon (C), nitrogen (N), and oxygen (O) were calculated to estimate the functionalization extents (Table 2) and the peak of C and N was reported to show the curve evolution due to the intermediate and final surface modifications (Fig. 3).

**Table 2:** Mean of atomic concentrations obtained with XPS analysis. Comparison among untreated and chemically modified PI films.

	PI	PI_v	PI_a	PI_v+IKV	PI_a+IKV
C (%)	77,31	74,82	68,67	74,6	68,57
N (%)	6,89	10,06	12,39	8,02	12,1
O (%)	15,82	14,4	17,45	15,92	17,94

After the introduction of vinyl groups on the PI surface, a decrease in the carbon atomic concentration and a parallel increase in the nitrogen one were observed and quantified for PI_v, and confirmed, as in previous works^34^, the success of the first step functionalization. The second step functionalization, obtained as a covalent bonding of IKV peptide to the substrate, led to a relevant decrease of the N atomic concentration because of a possible masking of the N atoms by the numerous C ones of peptide backbone. Furthermore, an increase of the oxygen percentage was observed (Supplementary Figure S2). These results were also expected due to the peptide composition, and in particular to the serine residues. In fact, PI_a showed a most significant change in nitrogen concentration and demonstrated the successful introduction of amino- reactive groups after the polyimide treatment. Nevertheless, contrary to what we found for the other approach, in this case the addition of IKV didn’t induce any remarkable change in the C or N atomic concentration suggesting a reduced efficacy of the peptide conjugation.

These outcomes identified PI_v+IKV as the more successful approach between the two. Beyond the more effective surface functionalization induced by the PI methacrylation, the thiol-(meth)acrylate Michael addition^51^ led probably to a favoured and stable peptide conjugation when compared to the standard peptide reaction.

### *In vitro* study

In order to evaluate the materials biocompatibility and cell response to the different substrates, PC12, primary Schwann cells (SCs), and fibroblasts were seeded on the engineered samples and on the relative controls. Collagen and Poly-D-Lysine-coated (respectively for PC12 and SCs) and untreated (for fibroblasts) standard plastic culture plates were chosen as positive controls, while untreated glass coverslips as the negative one for each experiment.

**Figure 4:**
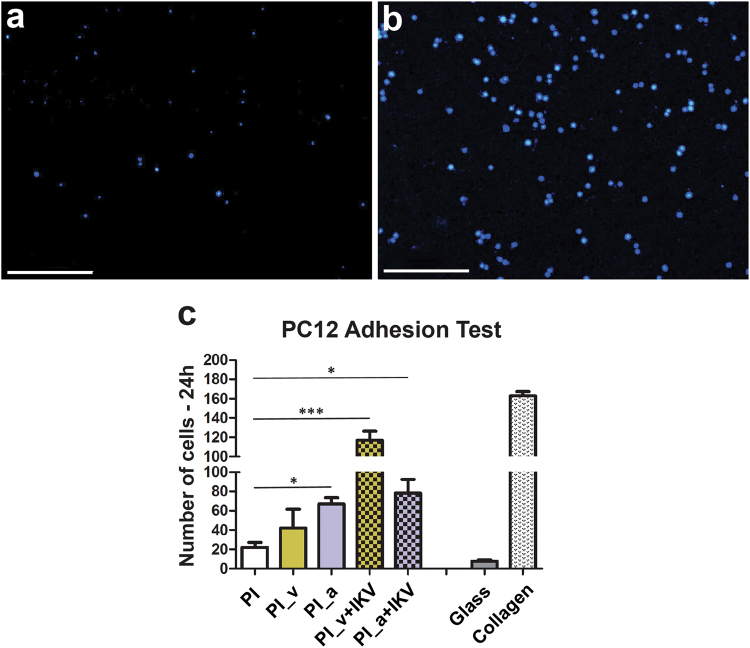
Comparison of PC12 cell adhesion on different substrates. (**A**,**B**) Nuclei stained with DAPI. (**A**) PI; (**B)** PI_v+IKV. The magnification is 10X and the size bar is 100 µm. (**C**) Cell count means are reported, and error bars correspond to the calculated SEM. A statistical analysis was performed using one way ANOVA (Dunnet’s Vs PI) and significant differences between the populations means are reported (*P < 0.05, ***P < 0.01).

**Figure 5:**
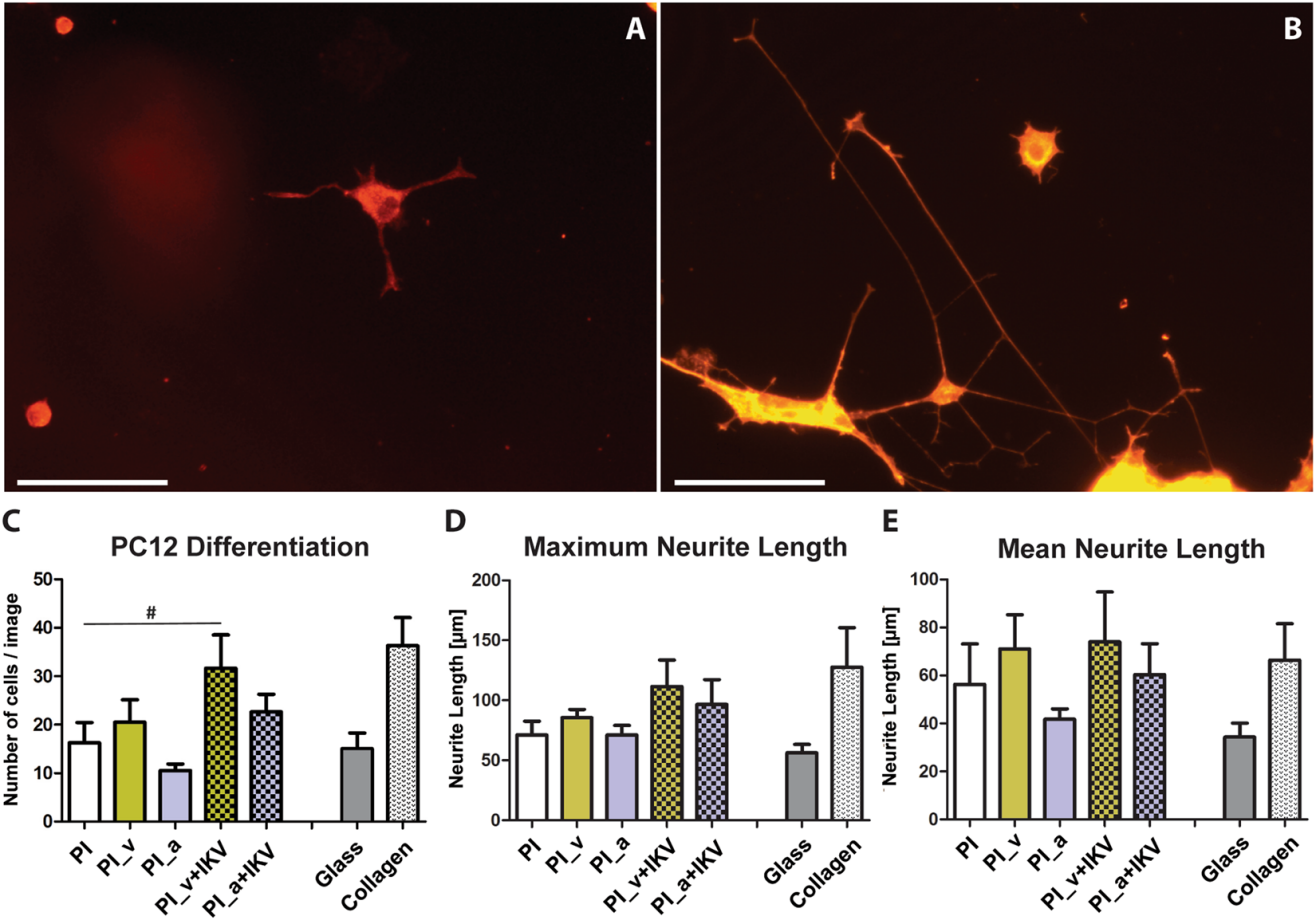
Comparison among different samples in terms of cells differentiation and neurite sprouting, after the addition of NGF to the PC12 culture. (**A**,**B**) Actin cytoscheleton stained with phalloidin and imaged with TRITC filter at 605 nm. (**A**) Untreated PI; (**B**) PI_v+IKV. The magnification is 20X and the size bar is 50 µm. (**C**,**D**,**E**) Quantitative results and statistical analysis to evaluate the number of differentiated cells. The mean of cell number per micrograph are reported and error bars correspond to the calculated SEM. A statistical analysis was performed using the t-test, and significant differences between the populations’ means are reported (P < 0.05).

PC12 cell adhesion was quantified to assess the short-term (24 h) response of cells to different samples (Fig. 4 and Supplementary Figure S3); moreover, cell morphology, spreading and NGF-stimulated neurite sprouting were measured to estimate cell differentiation upon different surfaces (Fig. 5).

Outcomes showed an increased density following the polyimide surface modifications steps, culminating with remarkable results for PI_v+IKV (P < 0.01 Vs PI, Dunnet’s test) due to the improved cell interaction with the IKV on the surface (Fig. 4).

Alternatively, the PC12 cells were cultured and differentiated by NGF starting from 24 h after seeding. They were kept in culture and monitored for 7 days and then fixed and stained for subsequent image analysis with NeuronJ. The contribution of each PI modification on cell differentiation into a neuronal phenotype was estimated (Fig. 5C) and the number and length of PC12 protrusions were quantitatively evaluated, as described, as maximum and mean neurite length (Fig. 5D,E). The results showed neuron-supporting properties up to 7 days in culture, especially for PI_v+IKV and PI_a+IKV.

Since PC12 cells scarcely adhere and differentiate upon substrates, if not supported by coatings as collagen or laminin, these results strongly support the notion that the IKV-modified polyimide films have an improved biocompatibility, compliance and no apparent toxic effects.

Furthermore, the outcomes on PI_v and PI_v+IKV confirmed the results found in the short-term adhesion analysis, resulting in a positive trend for cellular long-term attachment, differentiation and neurite sprouting for both the sequential surface modifications, thus leading to assume that vinyl groups and vinyl-bound IKV contribute to improve surface compliance for PC12 differentiation (Fig. 5C). Statistical analyses, in particular the t-test one performed between the untreated PI and the other samples, confirmed a significant difference for PI_v+IKV (^#^P < 0.05).

While maintaining the same positive trend, the cell response in presence of amino-groups resulted in a reduced capability of PI_a to support PC12 long-term adhesion, differentiation and neurite sprouting, when compared to the other samples. Nevertheless, the results for PI_a+IKV confirmed the IKV conjugation to positively affect also in this case the neural cell response, underlining the positive effects on cell viability of the IKV bonding with polyimide.

**Figure 6:**
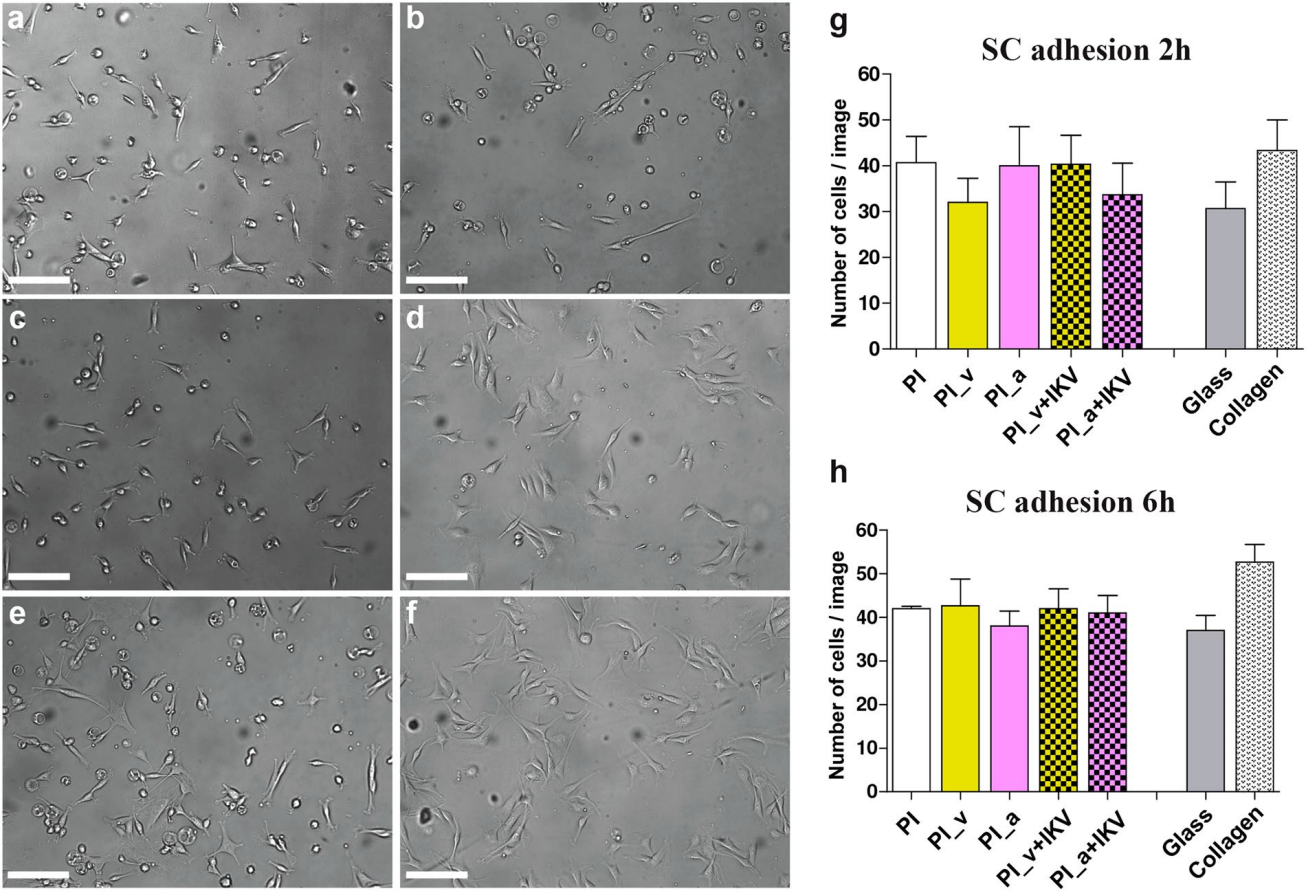
Short-term Schwann cells adhesion evaluation. (**a**,**b**,**c**,**d**,**e**,**f**) Visible light images of SCs after 6 h from seeding upon different substrates. (**a**) Glass coverslip; (**b**) PI; (**c**) PI_v; (**d**) PI_a; (**e**) PI_v+IKV; (**f**) PI_a+IKV. The size bar is 100 µm. (**g**,**h**) The histograms represent the number of cells per image calculated 2 h after seeding (**g**) and 6 h after seeding (**h**) respectively, shown as means with the error bars representing the related SEM. The statistical analysis was performed using a one-way ANOVA (Dunnet’s Vs PI) but no significant difference in cell density across the different substrates was found.

In order to assess the trend of attachment and growth of peripheral glia Schwann cell on the engineered substrates, primary rat SCs were seeded on the different samples. Their adhesion was quantified after 2 h and 6 h, and evaluated in terms of cell density upon the substrates (Fig. 6). Results evidenced a comparable SCs response across the different samples. The absence of significant differences during this short-term measurement was expected as described in a previous work^52^ that demonstrated a weaker effect of the IKVAV peptide on cell adhesionwithin 24 h in culture.

With the aim of studying SCs viability and growth over a longer period, a subsequent assessment was performed by a cell proliferation test performed after 24 h and 72 h respectively.

**Figure 7:**
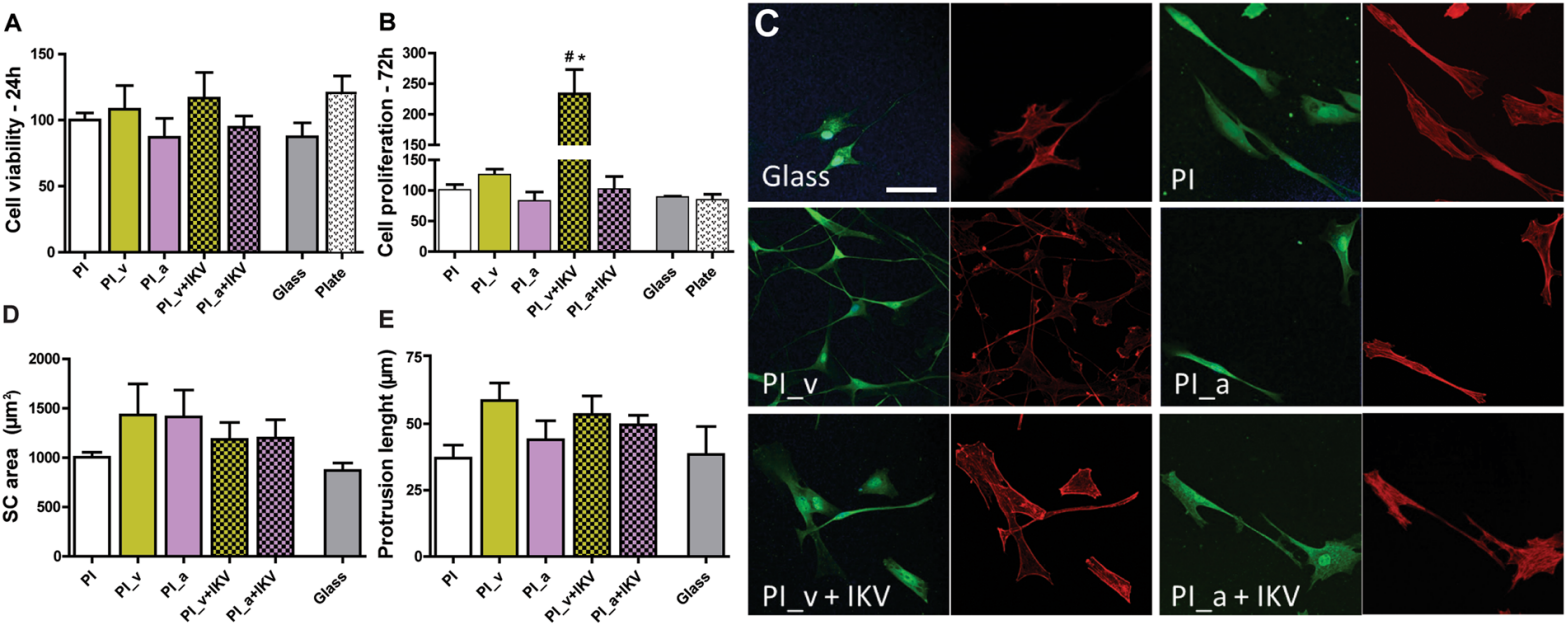
Comparison of SC cells viability and growth on different substrates. (**A**,**B**) Cell viability and proliferation after 24 and 72 h. (**C**) SCs cultured on different substrates and immunostained for S100 (green) and actin fibers (red); scale bar is 50 µm. (**D**,**E**) Morphological analysis of SCs: SC area (**D**, in µm^2^) and protrusion length (**E**, in µm) are reported for different substrates and error bars correspond to the calculated SEM.

SC viability was comparable in all substrates after 24 h (P > 0.05) (Fig. 7A). After 72 h (Fig. 7B) the cell proliferation rate showed an increasing trend on PI_v treated substrates: in particular the SC growth was increased on PI_v+IKV (P < 0.05 vs. PI, Dunnett’s test). The IKV functionalization through amino group linkers did not show any improving effect on SC proliferation.

SCs were then cultured on the different PI substrates, immunostained for S100 (a SC marker) and actin fibers (Fig. 7C), then the cell morphological parameters of individual SCs were analyzed by measuring cell shape, elongation and differentiation. SCs showed similar morphology on the different substrates, with no changes in their area (Fig. 7D; P > 0.05) and elongation ratio (Table 3; P > 0.05).

**Table 3:** SCs Elongation ratio on different samples, expressed by mean value and SEM.

	Glass	PI	PI_v	PI_a	PI_v+IKV	PI_a+IKV
SCs elongation ratio	3,6 ± 0,2	4,5 ± 0,8	3,7 ± 0,3	3,2 ± 0,5	3,9 ± 0,4	3,5 ± 0,4

Furthermore, we quantified the sprouting from SCs by measuring the neuritic-like protrusions emerging from each cell, which *in vivo* are needed to envelope axons (Fig. 7C). The number of protrusions *per cell* was constant on all substrates (~2 for all substrates as shown in Supplementary Figure S4), and SCs were found to be mainly bipolarized on functionalized polyimide substrates, meaning that SCs were responding well on those surfaces. Finally, we observed that the protrusion length was not negatively affected by surface functionalization and indeed it showed an increasing trend on PI_v and PI_v+IKV substrates, even if not significant (Fig. 7E; P > 0.05). Overall, PI-functionalized substrates induced a bipolarization effect on SCs, without inhibiting the protrusion growth.

The *in vitro* biocompatibility study with SCs thus confirmed the results obtained with the neuronal PC12 cell model, suggesting again the PI_v+IKV as the more suitable coating film for further neural interface applications.

**Figure 8:**
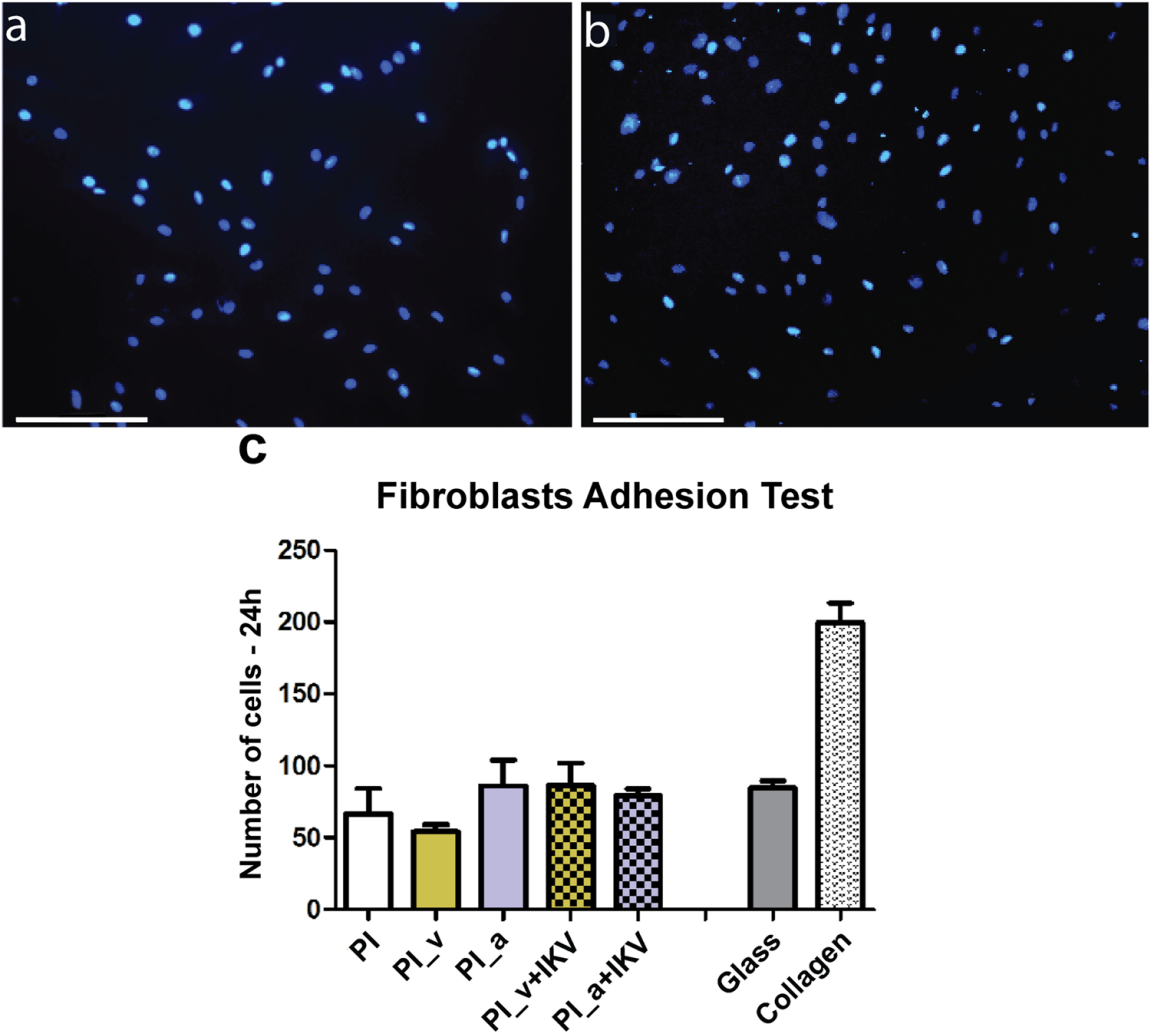
Comparison of fibroblasts adhesion on different substrates. (**A**,**B**) Nuclei stained with DAPI. (**A**) PI; (**B**) PI_v+IKV. The magnification is 10X and the size bar is 100 µm. (**C**) Cells number means are reported and error bars correspond to the calculated SEM. A statistical analysis was performed using a one way ANOVA (Dunnet’s Vs PI) and no significant difference was found.

Finally, we quantitatively evaluated fibroblast adhesion upon the different substrates (Fig. 8 and Supplementary Figure S5).

The results showed no evidence of a significant difference in cell response to functionalized polyimide substrates. For these cells, the film functionalization with vinyl-IKV is therefore not significantly improving the fibroblast adhesion. The significance of this outcome is crucial for the purpose of this work, and even more remarkable if compared with the neural and glial cells response. These cell types, usually less inclined to adhere and proliferate when compared with fibroblasts, demonstrated to better respond to PI functionalizations than NHDF cells, especially with PI_v+IKV. These results evidence how the modification of the polymide surface by the IKV peptide sensibly influenced this kind of cells response.

All these results combined, specifically indicate PI_v+IKV as a promising coating approach and encourage the use of this peptide-based biomaterial for *in vivo* testing and further neural engineering applications.

## Discussion

This work presented an advanced concept of polyimide coating, devised to improve the surface properties of the insulating thin-film component of a flexible invasive neural interface. The covalent conjugation of polyimide with a laminin-derived peptide was designed to increase the neuroelectrode coating biocompatibility and to promote the SCs response while hampering the fibroblast contamination of the surface. This approach was conceived to establish conditions and to validate a proof of concept able to improve the longevity of an implantable polyimide-based neural interface in the near future, especially if targeted to the peripheral nervous system.

Two peptide-based coatings, PI_v+IKV and PI_a+IKV, engineered to be easily implemented in the microfabrication process of a thin-film electrode and to be suitable for different designs (i.e., tf-LIFE, TIME, SELINE neural interfaces) were specifically developed not only to support neurons, but to also promote SCs response^15–17^ and to reduce the risk of fibroblast-mediated fibrotic encapsulation that usually insulates^14^ and compromises the performance of a neural interface^13^.

With this purpose, a laminin-derived sequence (CAS-IKVAV-S) was covalently conjugated to vinyl- and amino- groups exposed on functionalized polyimide sheets (PI_v and PI_a respectively). The morphological alterations (roughness, wettability and topography) and chemical changes (the presence of the bioactive molecules) induced on the polyimide surface were proved to significantly influence its interactions with several cell types.

*In vitro* analysis confirmed an improved biocompatibility and a good neural response for both the conjugation approaches, demonstrated by an overall increase in neuronal adhesion, neurite sprouting and peripheral glial cell viability. In particular, the PI_v+IKV films showed to promote PC12 adhesion after 24 h and neurite sprouting up to 7 days, and to support Schwann cell adhesion and proliferation together with good bipolar differentiation characteristics. On the other hand, the absence of comparable adhesion responses for fibroblasts lead also to assume for this biomaterial an enhanced polymer/fibroblast mismatch effect.

Since an ideal material implanted in the peripheral nervous system should promote neural cell and SCs viability and avoid fibroblast contamination^15^, the significance of these outcomes is crucial for neural invasive devices.

Aiming at the chronic stability of neural interfaces, technical solutions for biocompatible coatings that can be quickly transferred to clinic are highly needed: while many advanced and scientifically promising coatings have been developed and investigated so far, the real application of thin-film electrodes on human patients is still widely based on materials such as polyimide. The complexity that usually characterizes these strategies restricts, indeed, their portability and implementation on the manufacturing processes of the flexible electrodes. For this reason, we purposely designed an easy-to-implement approach for a quick translational applicability, and our findings show indeed the potential for a new generation of simple-to-attain and effective coating (see Table 4 for a comparison).

**Table 4:** Endpoint comparison among different solutions for invasive neural interfaces coatings.

	Approaches and/or fabrication techniques		*In vitro* biocompatibility		Fibroblast compliance
	Implementation complexity	Suitability	Neural support	Glial support	
Untreated polyimide	Low	High	Limited	Limited	High
Hydrogel for biomolecules release	High	Low	Good	Good	Reduced
Laminin-based coating	Medium	Medium	Good	Good	High
RGD -based coating	Low	High	Good	Good	High
IKVAV-based coating (other works)	Low	High	Good	Reduced	Reduced
**Present work**	Low	High	Good	Good	Reduced

The remarkable results related to PI_v+IKV, in particular, allowed to identify this solution as a promising strategy for developing highly biocompatible implanted neural interfaces and pave the way for subsequent *in vivo* investigations.

Moreover, the possibility to exploit the vinyl- and amino- modified PI to bind different peptides and biomolecules, or the application of this concept to other polymers (i.e., parylene), could open up interesting directions toward a whole new class and new generation of peptide-coated flexible invasive devices.

## Electronic supplementary material

Supplementary Information
