# Supplementary material for: Peptide-based coatings for flexible implantable neural interfaces
Source: Sci Rep. 2018 Jan 11;8:502. doi: 10.1038/s41598-017-17877-y (PMC5765121; doi:10.1038/s41598-017-17877-y)
Supplement: Supplementary file 1 — Supplementary Information [file 41598_2017_17877_MOESM1_ESM.docx]

**Supplementary Information**

Peptide-based coatings for flexible implantable neural interfaces

M. Righi^1*^, G.L. Puleo^2‡^, I. Tonazzini^3‡^, G. Giudetti^1^, M. Cecchini^3^, S. Micera^1,4*^

^1^ The BioRobotics Institute, Scuola Superiore Sant’Anna, Viale Rinaldo Piaggio 34, 56025 Pontedera (PI), Italy

^2^ Istituto Italiano di Tecnologia, Center of Micro-BioRobotics@SSSA, Viale Rinaldo Piaggio 34, 56025 Pontedera (PI), Italy

^3^ NEST (National Enterprise for nanoScience and nanoTechnology), Istituto Nanoscienze-CNR & Scuola Normale Superiore, Piazza San Silvestro 12, 56127, Pisa, Italy

^4^ Bertarelli Foundation Chair in NeuroEngineering, Center for Neuroprosthetics and Institute of Bioengineering (IBI)-School of Engineering, École Polytechnique Fédérale de Lausanne (EPFL), Lausanne, Switzerland

***Corresponding Authors**

Prof. Silvestro Micera: silvestro.micera@santannapisa.it

Dr. Martina Righi: [martina.righi@santannapisa.it](mailto:martina.righi@santannapisa.it)


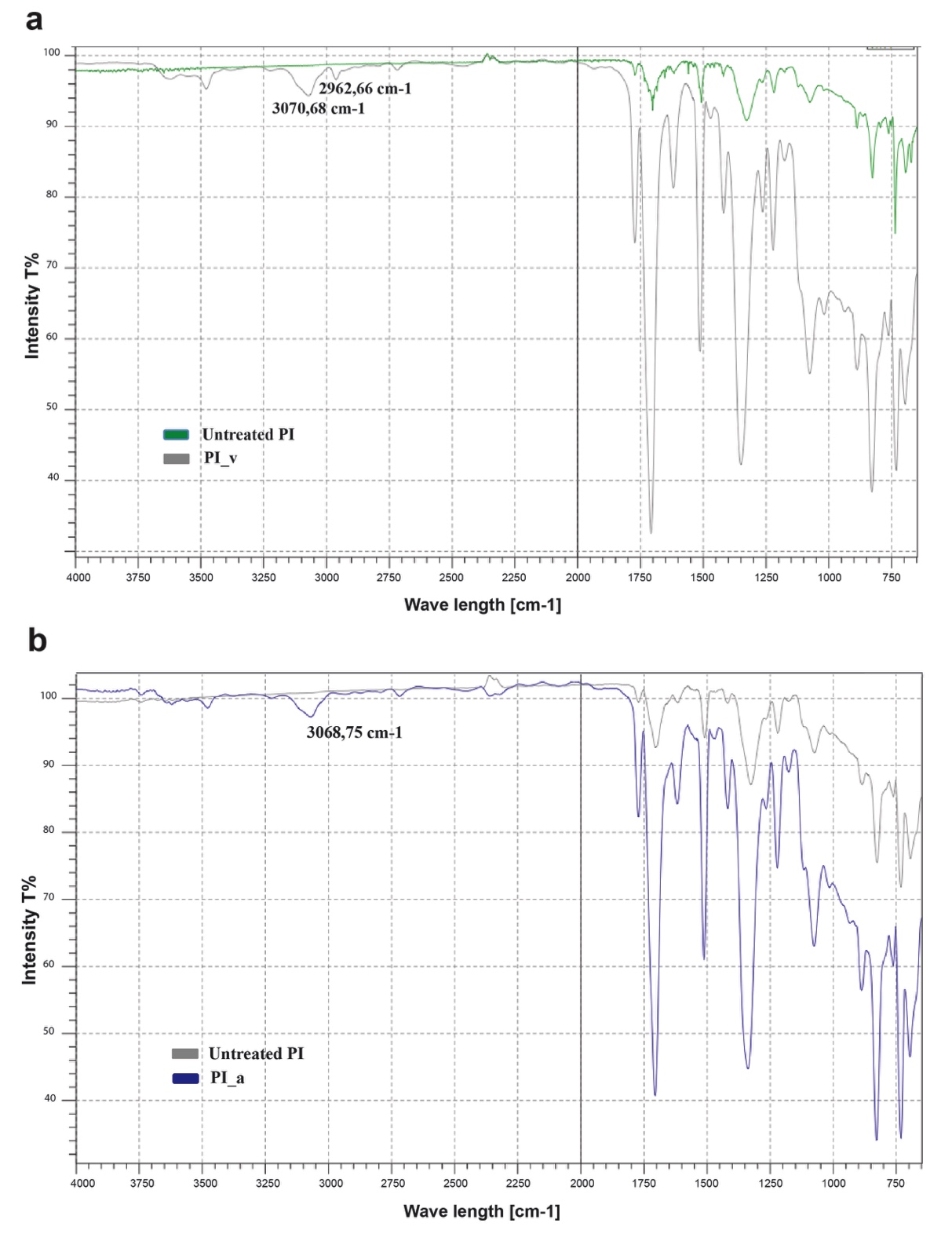


**Supplementary Figure S1.** FT-IR spectra of untreated and functionalized polyimide as collected on: a) PI and PI_v and b) PI and PI_a. Each sample was analyzed in transmission and spectra were compared in order to characterize the differences in surface molecular composition. The spectrum range was restricted to a selected window of 700-4000 cm^-1^.


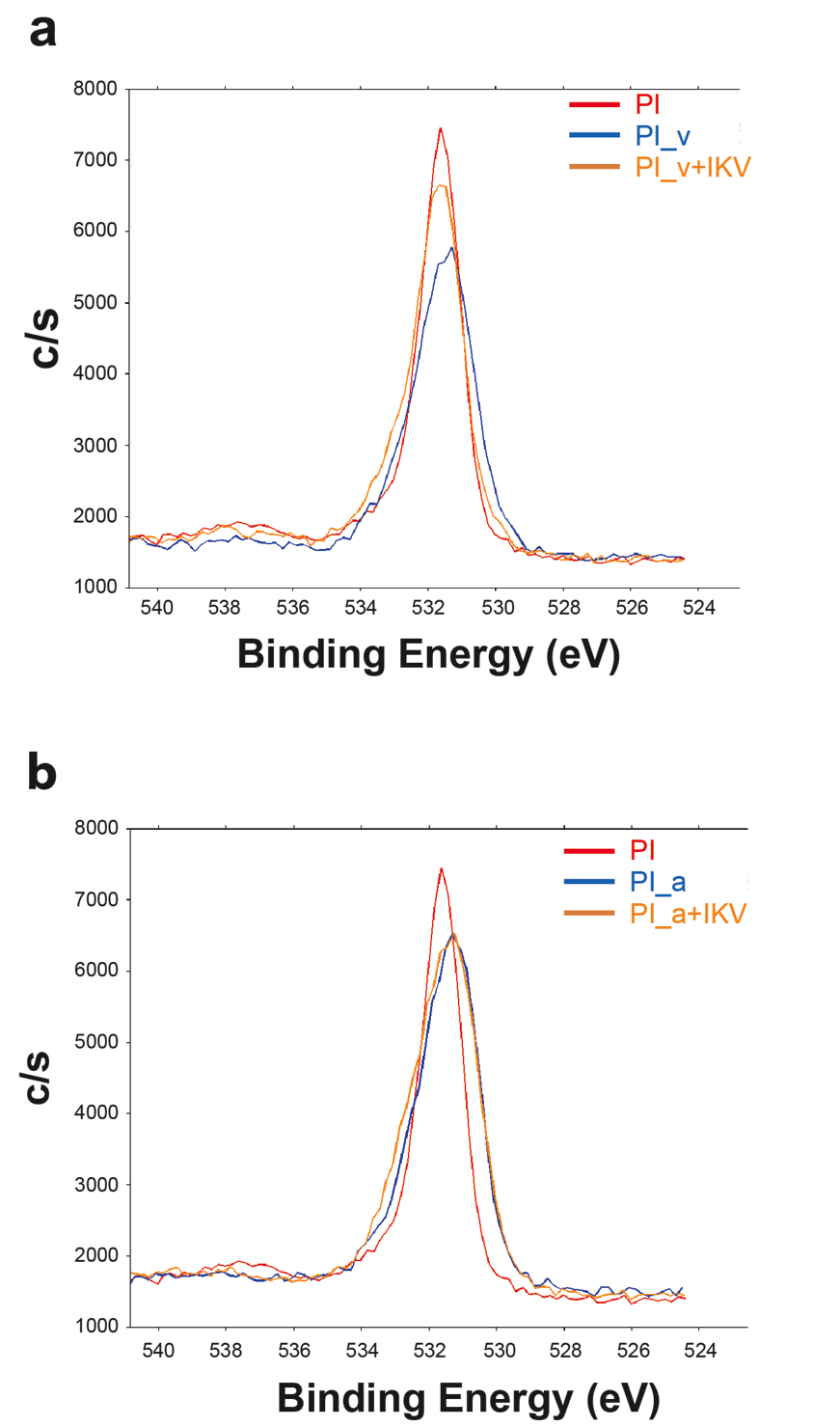


**Supplementary Figure S2.** XPS analysis on samples surfaces with Oxygen peaks and curves. Untreated PI was used as negative control and its surface composition compared with those of PI_v and PI_v+IKV (a) and with PI_a and PI_a+IKV (b).


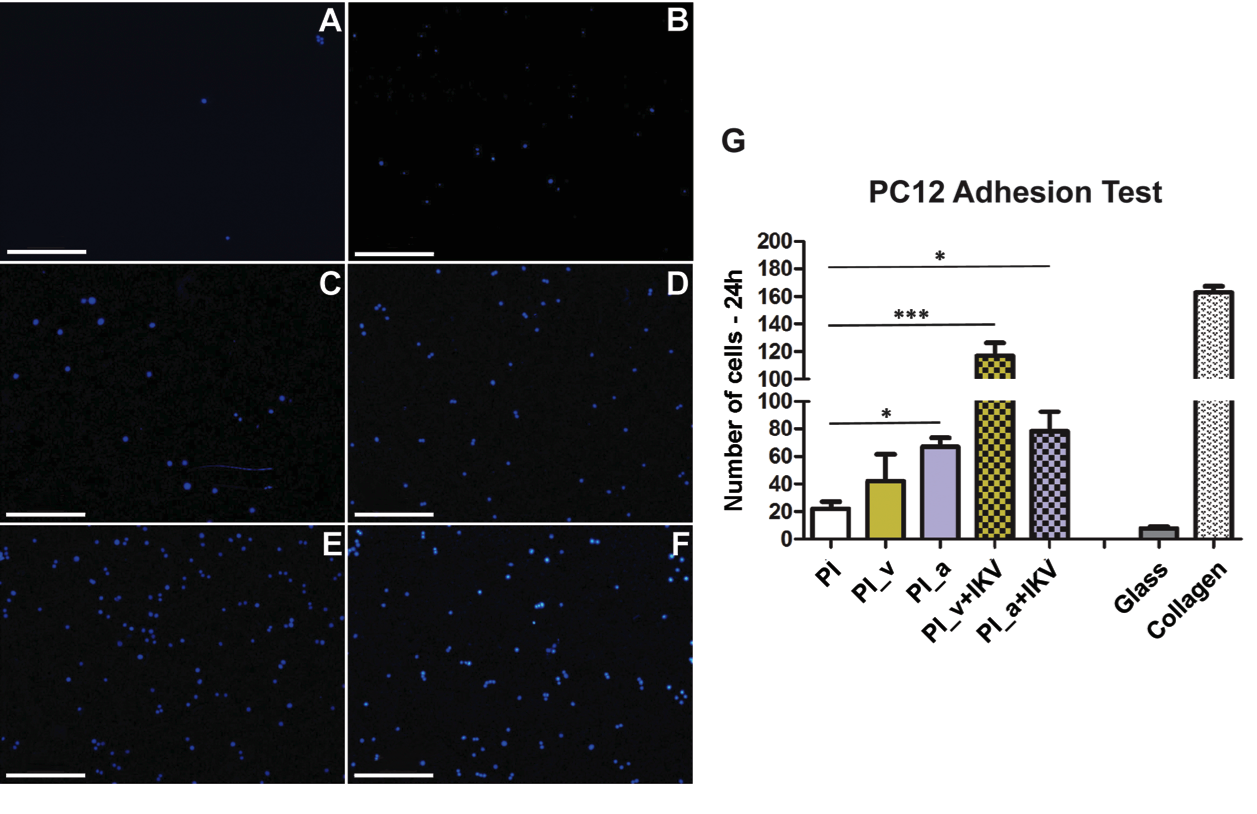


Supplementary Figure S3. Comparison of PC12 cell adhesion on different substrates. A, B, C, D, E, F: nuclei stained with DAPI. (A) Glass coverslip; (B) PI; (C) PI_v; (D) PI_a; (E) PI_v+IKV; (F) PI_a+IKV. The magnification is 10X and the size bar is 100µm. G: cell count means are reported, and error bars correspond to the performed SEM. A statistical analysis was conducted using one way ANOVA (Dunnet’s Vs PI) and significant differences between the populations means are reported (* P<0.05, *** P<0.01).


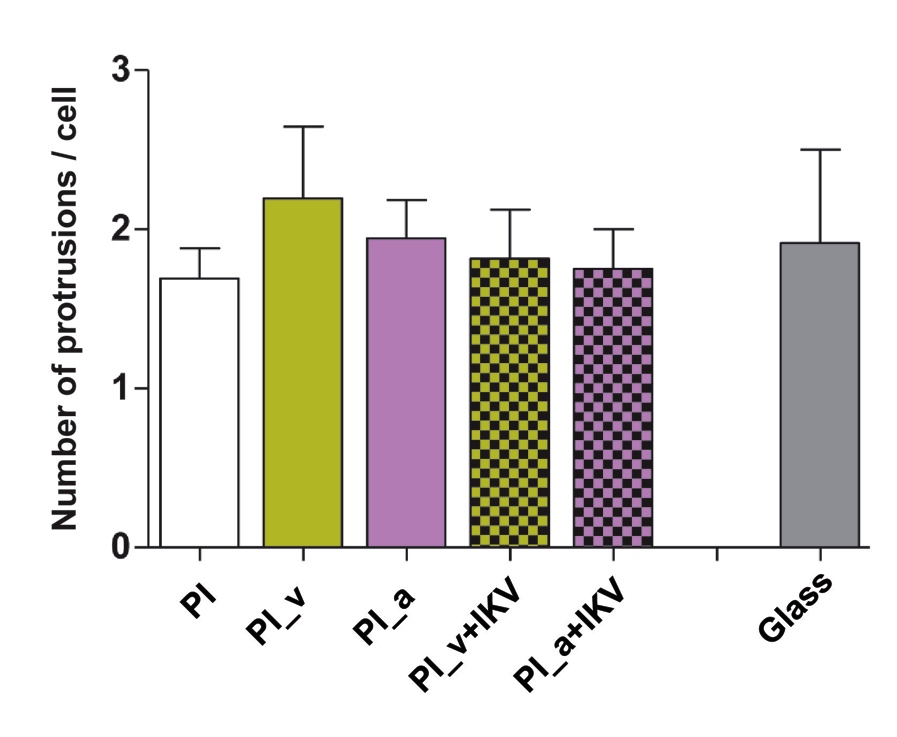


**Supplementary Figure S4.** Mean number of SC protrusions per cell reported for different substrates. The error bars correspond to the calculated SEM.


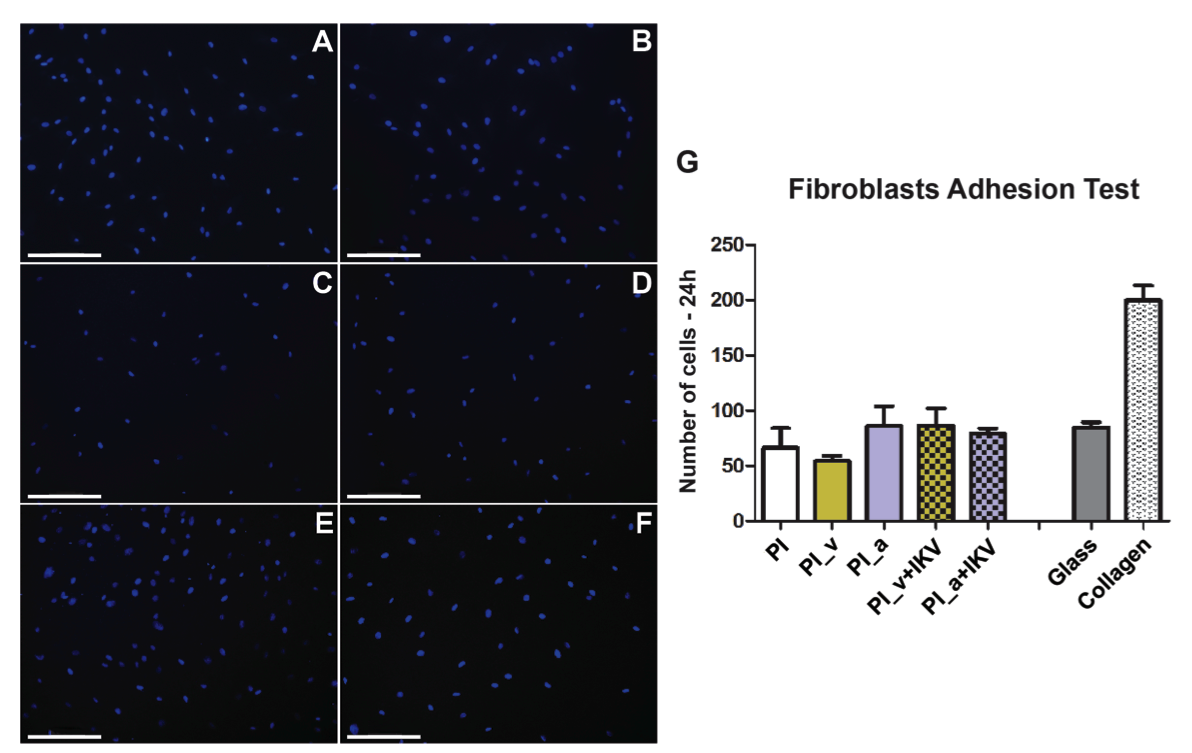


**Supplementary Figure S5.** Comparison of fibroblasts adhesion on different substrates. **A, B, C, D, E, F:** nuclei stained with DAPI. (A) Glass coverslip; (B) PI; (C) PI_v; (D) PI_a; (E) PI_v+IKV; (F) PI_a+IKV. The magnification is 10X and the size bar is 100 µm. **G:** cells number means are reported and error bars correspond to the calculated SEM. A statistical analysis was performed using a one way ANOVA (Dunnet’s Vs PI) and no significant difference was found.
